# Crossbow impact effect on clothing and potential associated injuries

**DOI:** 10.1111/1556-4029.70079

**Published:** 2025-05-19

**Authors:** Richard Critchley, Niall Patrick Hennessy, James Read, Rachael Hazael

**Affiliations:** ^1^ Cranfield Forensic Institute Cranfield University, Defence Academy of the United Kingdom Shrivenham UK

**Keywords:** ballistic injury, crossbow impact, fabric damage, fiber transfer, gelatine, non‐penetrating injury, penetrating injury

## Abstract

There has been an increase in crossbow use for hunting practices, sport target shooting, and criminal activity. In the UK, there is minimal legislation surrounding the crossbow. UK law states individuals must be over 18 to buy or possess a crossbow. To date, little experimental research has been conducted, with most of the research focusing on injuries resulting from crossbow incidents. The aim of this study was to ascertain what effect crossbow bolts would have against ordinary clothing worn by the public and how or if different combinations of clothing would fare differently against the bolts and if that could be of use to the forensic examiner. An 80 lb. Armex tomcat II crossbow was used with three types of clothing used in four combinations, along with a non‐clothed gelatine block. The results showed that all 10 bolts penetrated the non‐clothed gelatine block, T‐shirt, and polo shirt series, while nine bolts penetrated the hoodie/T‐shirt combination and only three penetrated the hoodie/polo shirt combination. Significant differences were highlighted between the mean penetration depths of the non‐clothed gelatine block and each clothing series, with the highest observed value being the thickest layer combination. Inspections of bolts and wound tracts revealed the presence of clothing fibers. The conclusions of this study demonstrated that this information can be utilized by forensic investigators and medical professionals as a source of trace evidence. Further research into crossbow effects on clothing would prove beneficial to increase the understanding of how the crossbow reacts with the environment.

**Highlights**

- Clothing provides a significantly higher level of protection compared with the non‐clothed gelatine block.
- Inspection of wound tracts and bolts revealed clothing fibers, primarily from the polo shirt and hoodie tests.
- Potential risk of internal injury exists due to non‐penetrating crossbow bolt impact with the target.

## 1 INTRODUCTION

The crossbow is believed to have originated in China as early as 400–300 BC, with written accounts from 341 BC describing its use in battle [1]. Bronze trigger mechanisms serve as evidence of their inception [1]. A form of crossbow is also believed to have been in use in ancient Greece, around 300 BC [1]. It was during the medieval period around 1100–1200 AD, however, where the crossbow became most popular and was used for hundreds of years [2]. The crossbow allowed the average person to become proficient in a shorter time than the longbow, due to requiring less skill to operate it. Crossbows had a much larger range than longbows and had stronger penetrating capabilities than the longbow, which led to a widespread use amongst Norman armies of the time [2]. They were initially made using wood, with later designs made with metal and early composite materials [3]. The crossbow was eventually phased out around 1700, due to the advent of gunpowder weapons, which were cheaper to manufacture and more potent than the crossbow. Crossbows have since experienced a surge in popularity, which could be attributed to the legislation surrounding them. In the UK, there is no requirement for a license to own a crossbow [4]; instead, the Crossbow Act 1987 [5] simply states that to purchase or possess a crossbow, the individual must be 18 years or older, compared with the rigorous checks and criteria that are carried out when an individual applies for a firearm license. In instances where a crossbow has a draw weight less than 1.4 kg, however, these systems do not have to follow this act. As a result of this, it is near impossible to predict how many people across the UK own a crossbow, compared with the known figure of around 730,000 registered firearms [6]. Interestingly, crossbow law varies significantly between the UK, Europe, US, Canada, and Australia. For example, across Europe, regulations vary with countries such as Germany and the Netherlands permitting ownership without a license but prohibit hunting [7]. Meanwhile countries like Sweden and Poland both classify crossbows as firearms, requiring licenses for possession while still prohibiting hunting [7]. Alternatively, Spain is one of if not the only European country which permits crossbow hunting but requires a License ‘E' [8].

In the US, the regulation on crossbows varies between states, with 29 states allowing unrestricted use of crossbows through all big game seasons. Some other states permit crossbows only during firearm season and Oregon banning crossbows entirely [9]. A list of states and their regulations can be found [10]. Canada allows crossbows to be purchased to individuals 18 and older and do not require a license unless the crossbow can be aimed and fired with one hand or crossbows which are shorter than 500 mm, then they are prohibited but some laws vary between provinces [11]. Australia is strict with their crossbow laws as many states classify them as prohibited weapons and require permits to use for target shooting [12]. Additionally, some states have banned pistol crossbows entirely due to their concealability and like with most countries you must be over the age of 18 to buy and own them [13]. From December 1, 2024, South Australia banned the use of bows and crossbows for hunting though target shooting is still legal [14].

Regardless of country of origin, it is important to note how crossbow systems compare with firearms in terms of projectile velocity, which owes to their threat. Typical handheld or pistol crossbows have a draw weight of around 80 lb. and can shoot bolts at approximately 55 m/s [15]. The more traditionally shaped, compound crossbows have a draw weight of around 150 lb. and shoot bolts at approximately 70–75 m/s [15]. In comparison, a 9 mm Glock handgun, for example, can shoot bullets at 375 m/s, and a 7.62 × 39 mm AK rifle can shoot bullets at 715 m/s [16, 17]. Although crime in the present day is more likely to see the use of firearms or knives, it is not completely uncommon to see the use of a crossbow. As recently as 2024, a crossbow was used in the murder of three women in Bushey, Watford [18].

There has been little research done experimentally on crossbows and their effects on different materials, objects, and the environment, compared with the extensive research undertaken on firearms [4]. This has led to difficulty for the forensic examiner or investigator when it comes to dealing with incidents where a crossbow has been used, but there has not been any recovery of a crossbow or bolts. There is also potential for incidents involving crossbow bolt injuries to be misidentified or interpreted as bullet wounds, especially when there is no evidence of crossbow use. A previous study by Randall and Newby sought to distinguish field tip crossbow bolt wounds from bullet wounds, due to the similarity between the two [19]. They concluded that bullet wounds were more circular and contained wipe‐off material that could be identified, compared with a field tip arrow that leaves more of an elliptical wound and no wipe‐off material. Chemical spot tests also revealed the presence of lead and/or copper in bullet wounds, compared with none in arrow wounds [19]. Other studies by Grellner et al. and Rogers et al. have looked at the injuries, and in some cases deaths, caused by crossbow bolts, be it intentional (murder or suicide) or accidental [20, 21]. Rogers et al. describe a case where an elderly man was found dead in his hospital bed, with a 17‐inch crossbow bolt embedded in his chest [21]. It had penetrated his heart, aorta, and part of his left lung before exiting the body and partially penetrating the mattress below. He had been killed by his son as a form of euthanasia. Grellner et al. report a case of suicide whereby the victim killed himself in the bathtub using a crossbow [20]. The bolt struck the man in the roof of his mouth and passed through part of his brain. He died because of central regulatory system collapse [20]. Although, similarly to gunshot wounds, crossbow injuries are survivable depending on where the region of damage is and the immediacy of care, as highlighted by Chang and Hsee [22]. While it is important to understand primarily how the body reacts to crossbow injuries, it is also of importance to understand how they react with the environment around the body. A study done by Standbridge et al. showed that a common automotive vehicle window was completely penetrated by crossbow bolts, leaving the occupants at risk [23], while the most recent study by Read et al. exposed various protection systems to differing crossbow bolt geometries [4].

An area that has not been researched in much detail is clothing and its effect against crossbow bolts. There has been a slightly similar experiment done in the past on the effect of different arrowheads fired from a bow against loose and tight clothing by MacPhee et al. [24]. The study looked at four different types of arrowheads: field tip, blunt, judo, and broadhead, and what effect they had on the two sets of clothing, which were T‐shirts and jeans. The aim was to see how this affected the penetration depth of the arrow. The study concluded that clothing compared with non‐clothing was able to reduce penetration of all arrow types. The penetration reduction of the clothing was dependent on the type of arrow used, but the study showed that the loose clothing was penetrated less. The loose jeans experienced the smallest penetration values due to their fiber makeup and tight weave. However, this study focused on arrows and archery systems as opposed to crossbow and bolts. Robertson et al. highlight the methods by which textiles sustain damage, alluding to failure [25, p. 71]. They include cuts from sharp instruments and punctures caused by impact from a blunt instrument with a pushing force. The other important one to note is a tear, which is damage caused by a pulling force [25, p. 71].

There has also been some research done on the ballistic effect on clothing and how it influences injury. Studies by Stevenson et al. [26, 27] found that the more layers being worn, the more likely it was for the gunshot wound to require a complicated surgical intervention, thus being more severe.

From the limited literature available, it is evident that further research is required to support law enforcement and the forensic practitioner in ascertaining what occurs when bolts impact individuals wearing a selection of clothing layers.

## 2 MATERIALS AND METHODS

### 2.1 Crossbow and bolts

**FIGURE 1:**
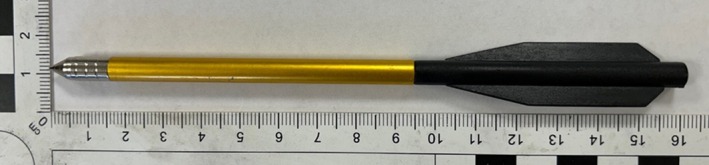
16.5 cm/ 6.5″ field‐tipped crossbow bolt used in experiment.

An Armex Tomcat II self‐cocking pistol‐style crossbow, with a draw weight of 80 lb, was used herein [28]. The bolts (defined as a dart like projectile used by crossbows) were 6.5″ (16.5 cm) in length with a mass of 9.5 ± 0.1 g and comprised an aluminum shaft, a steel field tip point, and a plastic fletching [29] (Figure 1). In this configuration, the crossbow is stated to discharge the bolts at a velocity of 195 fps (59.4 m/s) [28]. In all tests, the crossbow utilized the as‐sold iron sight and supplied string to represent a potential likely setup that a perpetrator may use.

### 2.2 Target

Three 10% ballistic gelatin blocks of dimensions 23 H × 23 W × 52 L cm were created as per method [30]. Upon removal, blocks were cut in half using a sharp blade to create six half blocks of dimensions 23 H × 23 W × 26 L cm. One block was used as a control, while four blocks were used per test series.

### 2.3 Clothing layers

**FIGURE 2:**
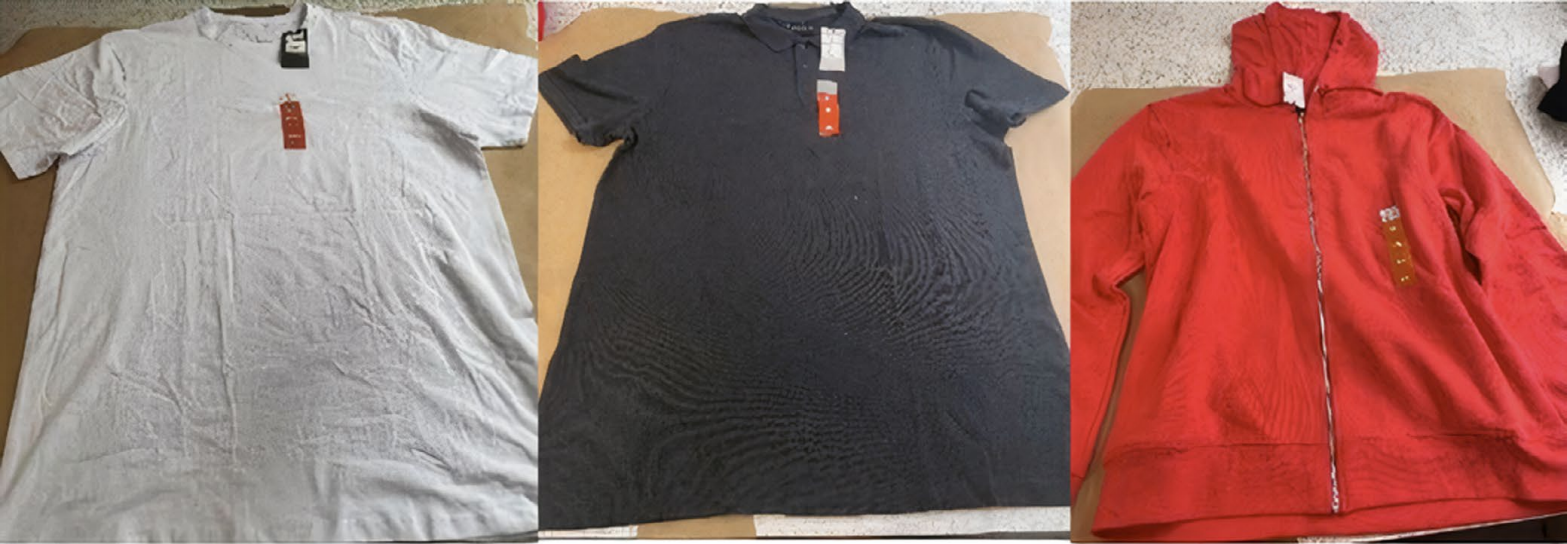
Clothing used in experiment L‐R: T‐shirt, polo shirt, hoodie.

Five‐test series were conducted exploring four clothing configurations alongside one baseline (Table 1). For each test series, ten shots were conducted, resulting in a total of 50 shots being completed. Clothing was purchased from Primark, UK, and was unused before firing. The garments consisted of a polo shirt (100% cotton, plain weave construction), a T‐shirt (95% cotton, 5% elastane, knitted construction), and a hoodie (50% cotton and 50% polyester, knitted construction) (Figure 2). Clothing was chosen to provide a representation of what the public could be wearing when involved in an incident. Due to its function, hoodies were tested in two‐layer configurations with either a T‐shirt or polo shirt underneath. To simulate typical clothing wearing, clothing layers were draped over the gelatin block (GB).

**TABLE 1:** Clothing configurations used throughout testing and layer thickness in mm.

Series	Target clothing combinations	Clothing layer thickness (mm)
1	Gelatine block (GB)	–
2	GB + T‐shirt	0.8
3	GB + T‐shirt + Hoodie	0.8 + 1.3
4	GB + Polo shirt	0.8
5	GB + Polo shirt + Hoodie	0.8 + 1.3

### 2.4 Experimental setup

Testing was conducted using the Small Arms Experimental Range at the Defense Academy of the UK, Shrivenham. The crossbow was set up at a height of 1.3 m and secured in place using a vice, while the target was placed 10 m down range from the crossbow at a height of 1 m. All shots in this study were conducted normal to the target.

**FIGURE 3:**
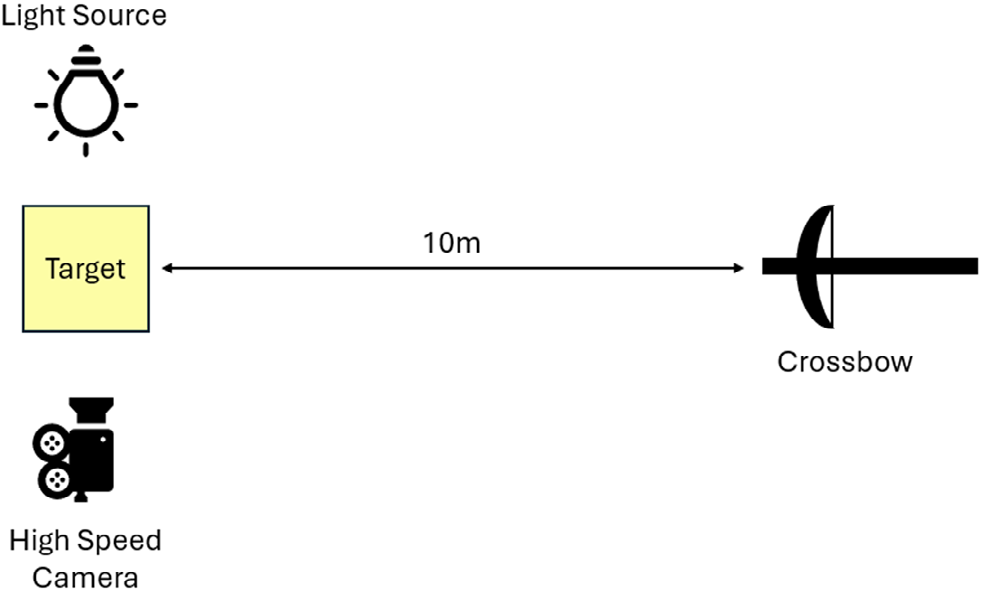
Diagram representation of experimental setup.

Once the target was placed, to simulate typical clothing wearing, clothing layers were draped over the gelatin block (GB), leaving the sides exposed to enable high‐speed video (HSV) usage. The HSV used herein was a Phantom V12 captured at 7900 FPS in 1024 × 768 resolution, positioned perpendicular to the GB, with a high‐powered light source opposite to illuminate the GB internals. Figure 3 provides a schematic representation of the setup. Prior to any testing, the crossbow was calibrated utilizing an attachable red dot holographic sight to ensure a central impact.

For each test, the crossbow was cocked, and a new bolt was loaded manually before firing remotely using a slack line attached to the trigger and pulled from within the safety room, where bolt velocity was captured using Weibel W700 doppler radar, where a mean impact velocity of 54.6 ± 0.2 m/s was noted.

Following impact, bolt penetration depth was calculated by measuring using a hand rule (error ± 0.1 cm) how much of the bolt shaft protruded from the GB minus the total length of the bolt. Wound tract inspection was completed by cutting through the GB using a sharp knife and carefully removing bolts before analyzing the tracts for any remaining evidence, such as clothing fibers or bolt fragments. The crossbow bolts were examined after removal using a magnifying glass (3×) to inspect for damage and remaining clothing fibers. Finally, the clothing was inspected using a magnifying glass (3×) to assess damage on the front and rear surface of the clothing layer(s).

## 3 RESULTS

### 3.1 Penetration data

**FIGURE 4:**
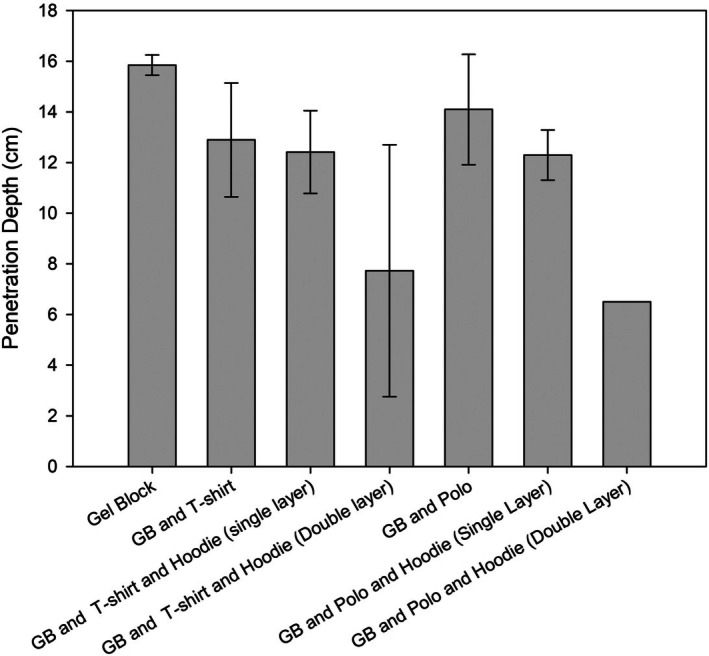
Comparison of mean penetration values where all clothing layers demonstrate a reduction in mean penetration.

Table 2 shows all penetration depths recorded over the five‐test series where no instances of perforation were noted. Overall impact velocity was found to be highly repeatable with a mean impact velocity of 53.05 ± 0.36 ms^−1^. In all tests against the GB baseline, penetration was found to be highly repeatable with a mean penetration depth of 15.85 ± 0.40 cm. When single clothing layers were tested, both the T‐shirt and polo shirt series demonstrated 100% successful penetration with mean penetration depths of 12.33 ± 2.25 cm and 9.3 ± 5.06 cm, respectively. Interestingly, when the hoodie layer was introduced, a change in behavior was observed. As aforementioned, the hoodie series was split into two subcategories of the hoodie being double (tests 1–5) and single layered (6–10). Analysis of the double and single layups backed by a T‐shirt found mean penetrations of 6.18 ± 5.52 cm and 12.4 ± 1.63 cm, respectively. It should be noted, however, that for the double layer configuration, one bolt failed to penetrate as it was observed to rebound from the clothing layers (observations via high‐speed video). Alternatively, analysis of the double and single layups backed by a polo shirt found mean penetrations of 1.3 ± 2.91 cm and 4.92 ± 6.76 cm, respectively. Unlike the T‐shirt backed hoodies, penetration success was significantly reduced, with the double layer only being penetrated once (out of five tests) and twice for the single layer, with bolts once again bouncing off the clothing layers. A comparison of the mean penetration values between series can be found at Figure 4.

**TABLE 2:** Penetration depths recorded for all test configurations, where “–” indicates no penetration into the gelatine block.

Series number	Target combination	Penetration depth of shot (cm)										Mean (cm)	Standard deviation (cm)
		1	2	3	4	5	6	7	8	9	10		
1	Gel block	16.0	15.2	16.3	15.8	16.5	15.9	15.4	15.9	15.5	16.0	15.9	0.4
2	GB and T‐shirt	13.1	13.4	11.7	13.3	11.1	12.5	12.3	13.5	6.9	15.5	12.9	2.2
3	GB and T‐shirt and hoodie (single layer)	11.7	10.7	12.3	15.1	12.3	–	–	–	–	–	12.4	1.6
3	GB and T‐shirt and hoodie (double layer)	2.6	14.5	7.5	DNP	6.3	–	–	–	–	–	7.7	5.0
4	GB and Polo	14.0	15.1	14.7	8.7	12.8	15.6	12.3	14.1	16.2	12.3	14.1	2.2
5	GB and Polo and hoodie (Single layer)	13.0	DNP	11.6	DNP	DNP	–	–	–	–	–	12.3	1
5	GB and Polo and hoodie (Double layer)	DNP	6.5	DNP	DNP	DNP	–	–	–	–	–	6.5	–

### 3.2 Bolt inspection

**FIGURE 5:**
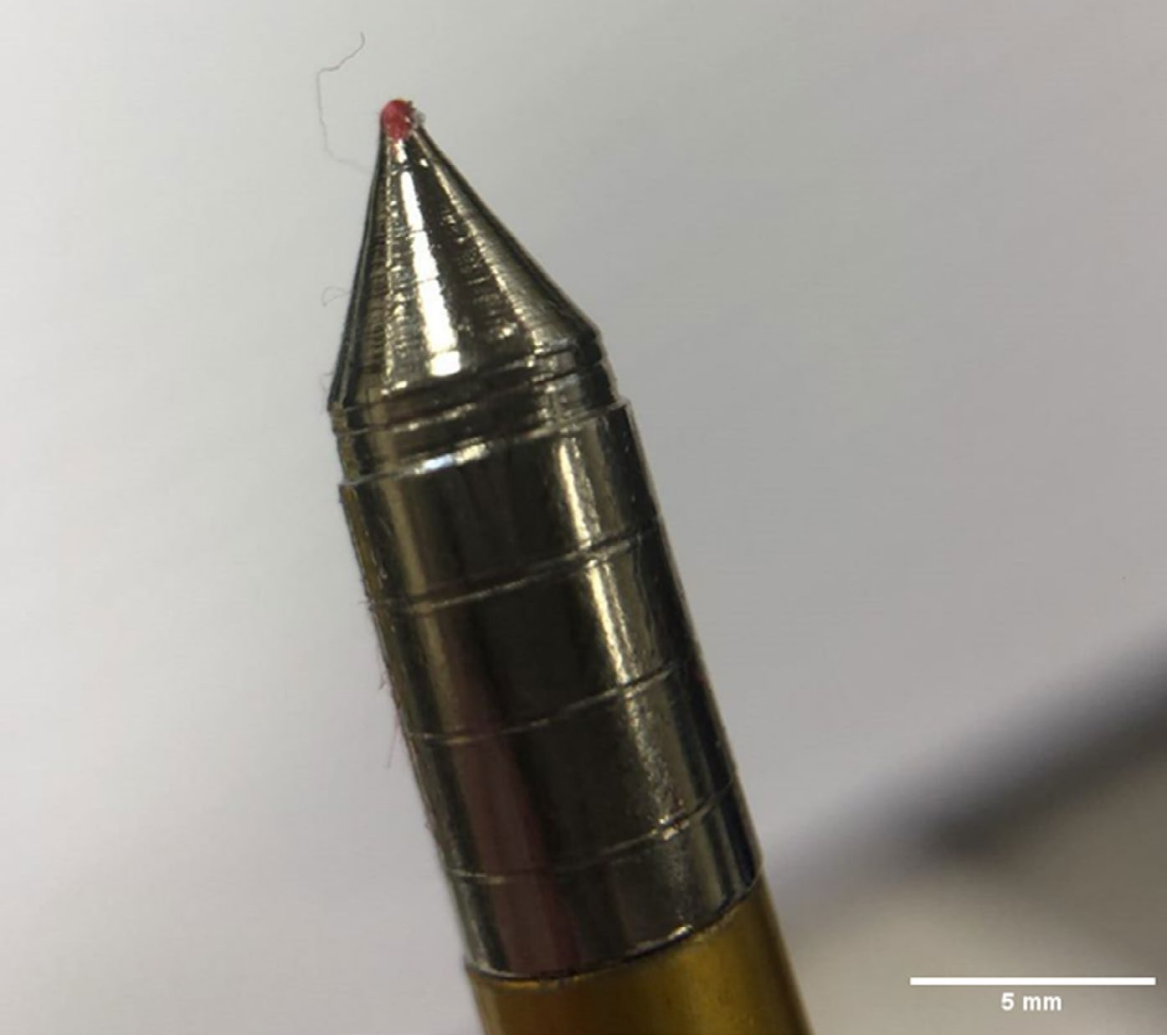
Red fibers on tip from hoodie from series 3.

Upon post‐recovery inspection of all 50 bolts, only a single bolt exhibited slight damage where the fletching had been chipped in the T‐shirt‐hoodie series, while no broken bolt pieces were found in any wound tracts. Interestingly, inspection of the bolts revealed the presence of fibers on approximately half of the bolts. In the gelatine block series, no fibers were present on any of the bolts, while in the T‐shirt series, four out of ten bolts had fibers from the T‐shirt remaining on them when removed. These fibers were found on three of the tips and one on the fletching. By contrast, all ten bolts fired in the T‐shirt‐hoodie series displayed red fibers remaining from the hoodie on the tip, but no white T‐shirt fibers were detected (see Figure 5).

**FIGURE 6:**
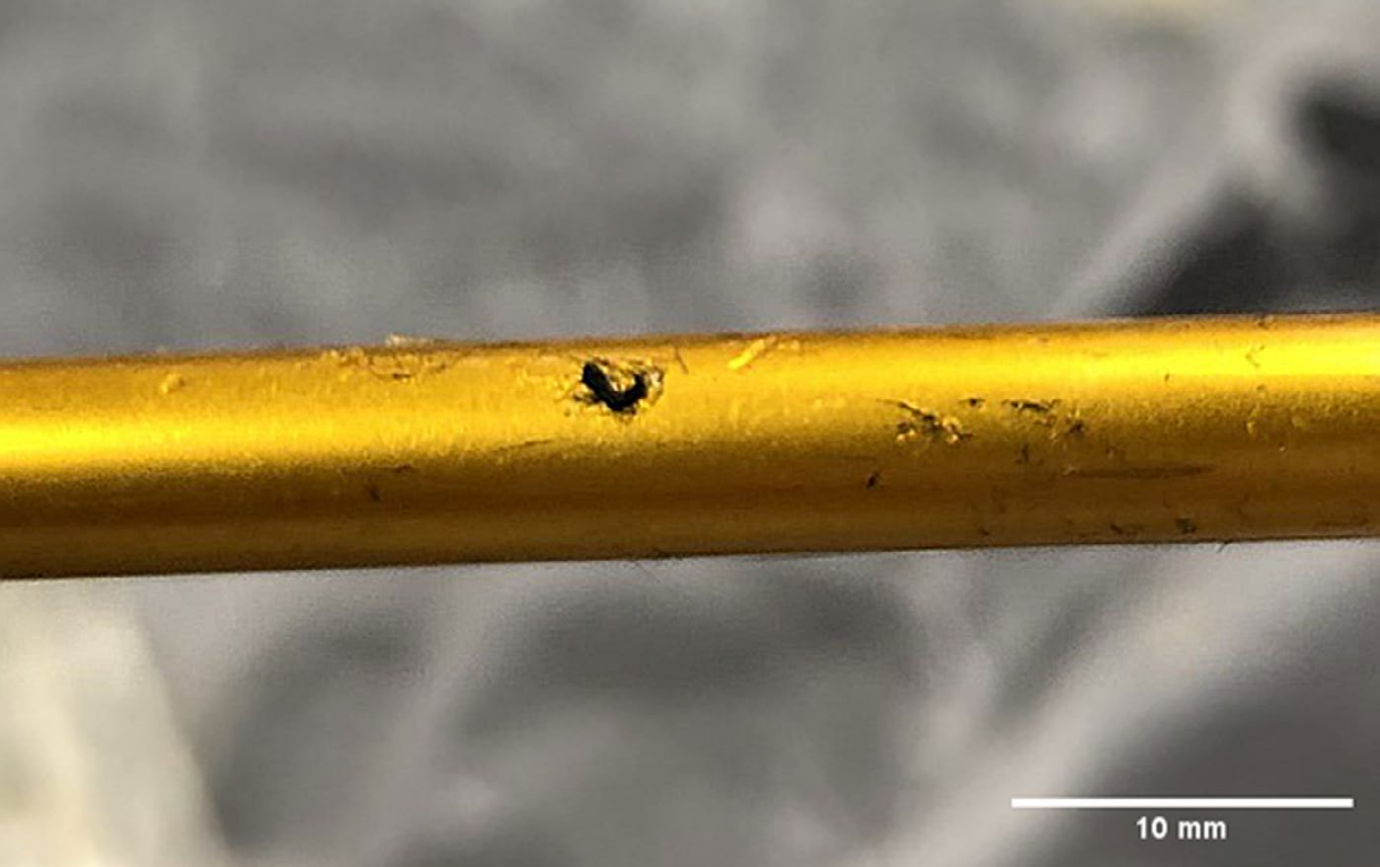
Black yarn on shaft from polo shirt from series 4.

The most prominent series in terms of fiber evidence was the polo shirt. All bolts in this series featured black fibers from the shirt, but unlike the other datasets, the fibers were all located on the shaft of the bolt instead of the tip (Figure 6). By contrast, the hoodie‐polo series did not exhibit any fibers on the bolts; however, this might be as a result of only three of the 10 bolts penetrating the clothing layers.

### 3.3 Wound tract inspection

**FIGURE 7:**
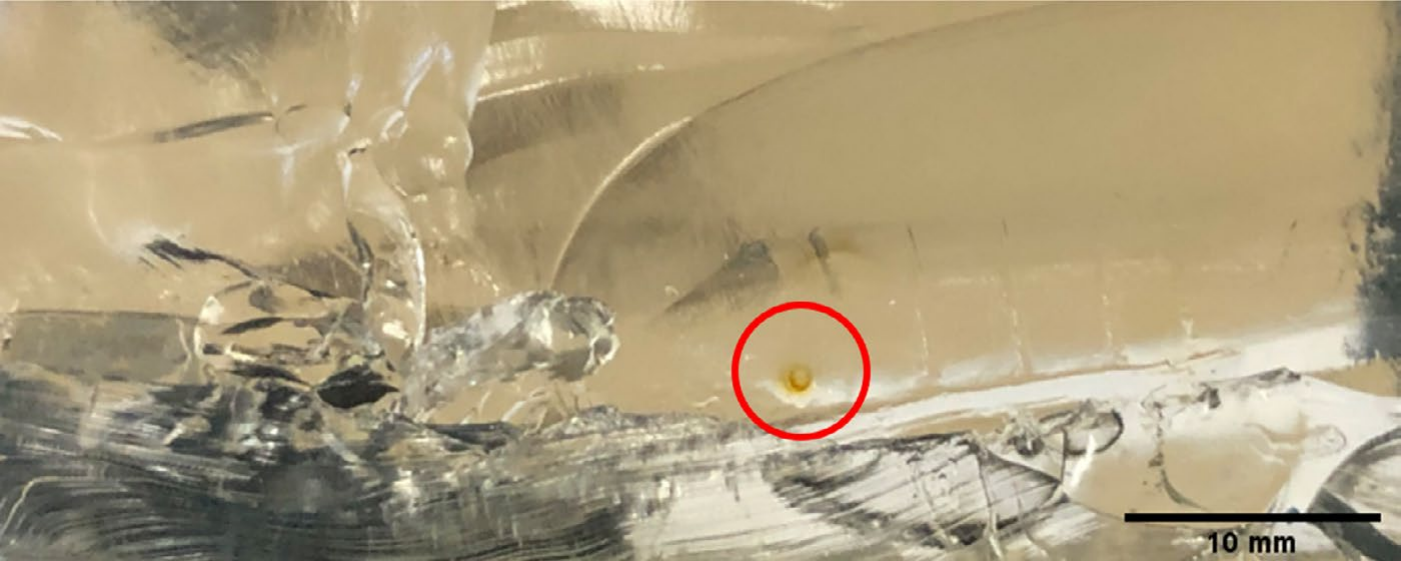
Observed discoloration, circled in red, in wound tract from series 1.

**FIGURE 8:**
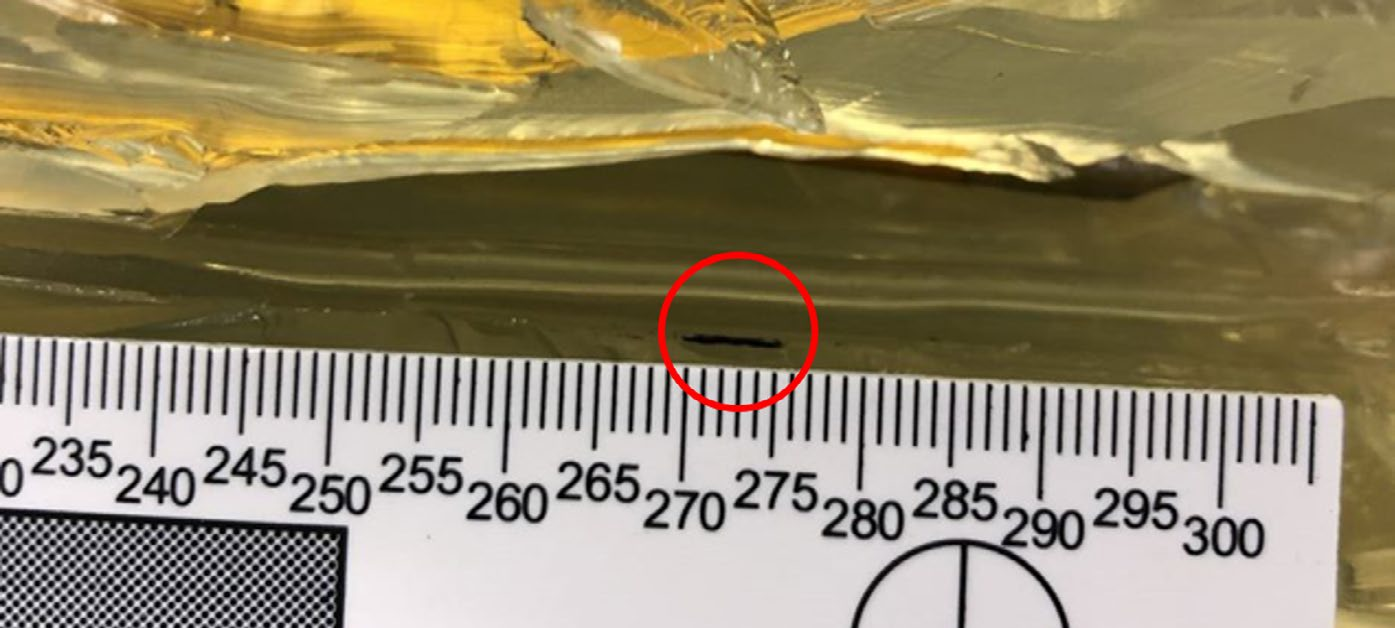
Circled in red—black yarn from polo shirt found within the gelatine block wound track.

Inspection of the wound tracts found no fibers in any of the wound tracts from the gelatine block series, as expected. No fibers were additionally found in the T‐shirt series, although three tracks contained discoloration (Figure 7). Of the nine wound tracts from the T‐shirt‐hoodie series, only two contained fibers, which were red from the hoodie. Alternatively, the polo shirt series exhibited either single or, in some instances, multi black fibers (Figure 8) in eight of the 10 wound tracks, while the polo shirt‐hoodie combination found red fibers from the hoodie in two of the wound tracks, with no evidence of polo fibers.

### 3.4 Clothing damage

**FIGURE 9:**
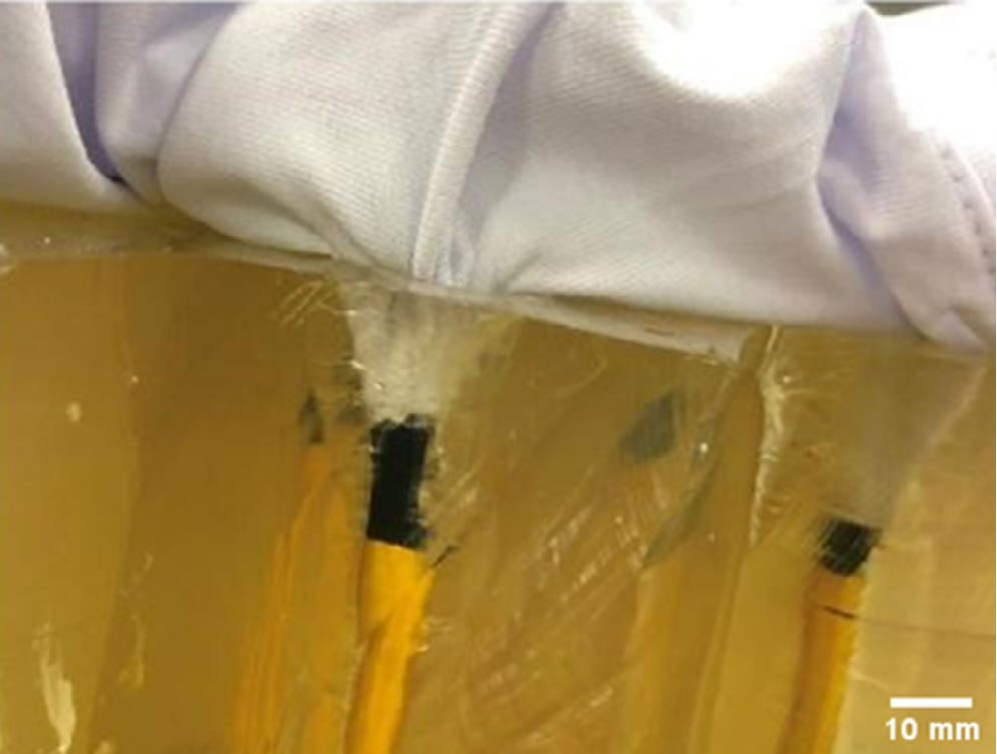
Illustrating pull‐in of T‐shirt into the wound tract by bolt, from series 2.

**FIGURE 10:**
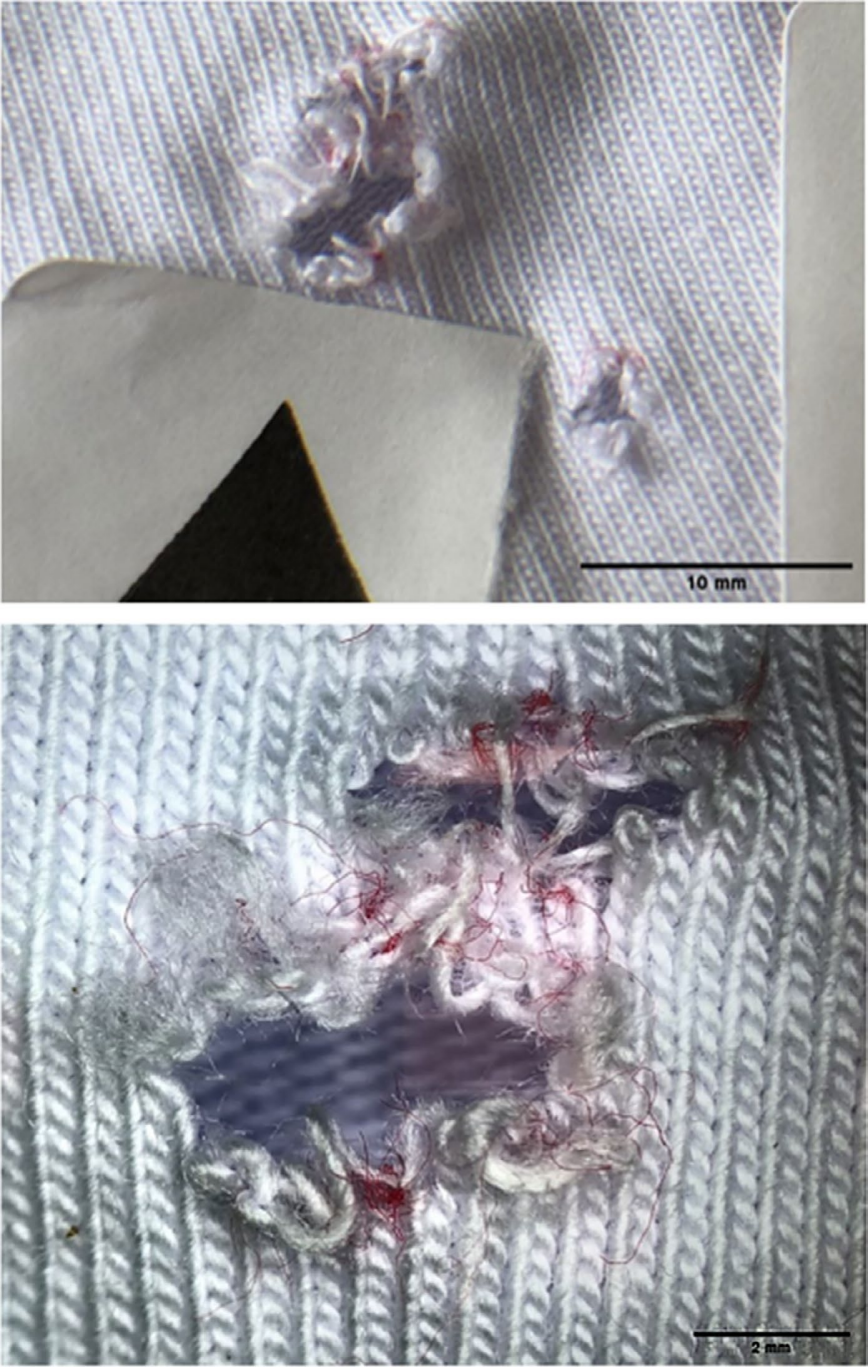
Deposited red fibers from the hoodie pushed through by the bolt onto the T‐shirt in series 3.

**FIGURE 11:**
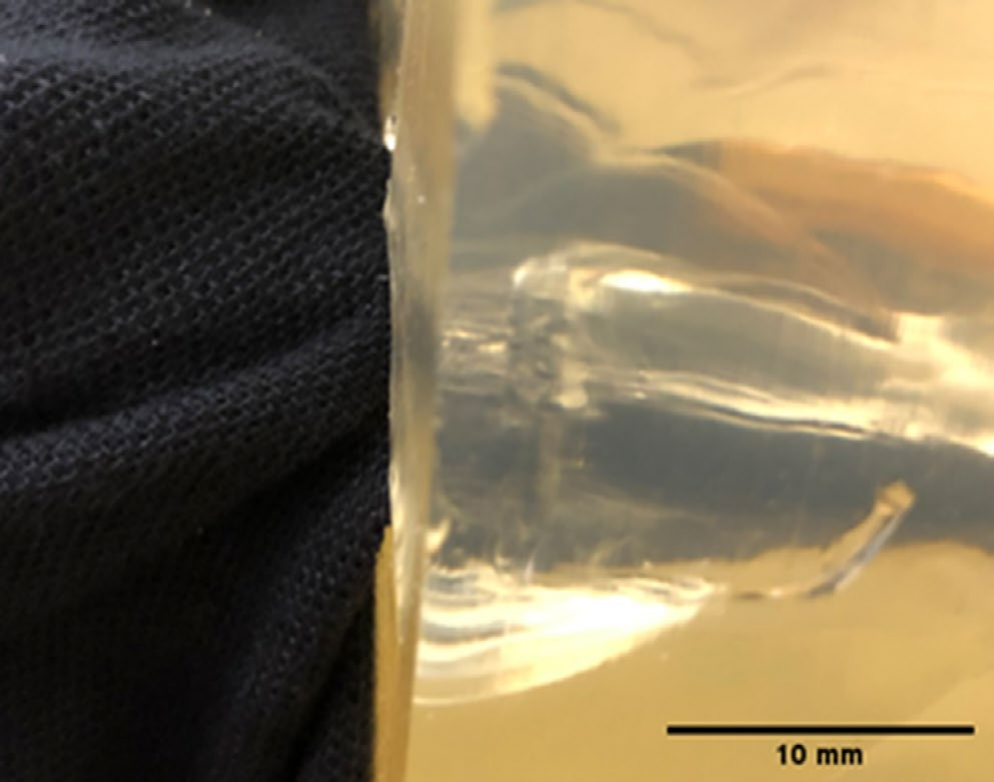
Illustrating pull‐in of polo shirt into wound tract by bolt, from series 4.

In terms of damage to clothing, the T‐shirt series featured some significant material pull‐in (described as the process in which a fiber experiences translation in the direction of the applied load, e.g., typically into a wound track) into the wound tracts, shown in Figure 9. The front surface impact point created by the bolts was subtle, ranging in size from 3 to 4 mm. There was some fiber pull‐out (described as the process in which a fiber experiences complete pull‐out when subjected to a load) visible on the rear surface as well. The rear surface damage ranged in size from 2 to 4 mm in diameter. For the hoodie/T‐shirt series, there were two halves: one with the hoodie double layered to capture high‐speed video and the second half where the hoodie was draped over the block and smoothed before impact. For the first half of the series, the front surface of the impact point of the hoodie resulted in fiber pull‐out and ranged in size from 3 to 5 mm in diameter. The rear surface damage was more pronounced, again featuring fiber pull‐out and ranged in size from 3 to 5 mm in diameter. For the second half of the series, the hoodie front surface impact point damage was again between 2 and 5 mm in diameter and featured fiber pull‐out. The front surface damage on the T‐shirt was more noticeable, with fiber pull‐out. The holes ranged in size from 1 to 5 mm in diameter. Each front surface impact hole had red fibers around the edge, pulled through from the hoodie by the bolt, shown in Figure 10. The rear surface damage holes all featured the red fibers apart from one, showing they had been pulled through the holes in both layers. The polo shirt series featured quite distinctive damage holes compared with the previous clothing. There was distinct material push‐in on the front surface, and these were all 5 mm wide apart from one, which was 8 mm in diameter. The rear surface damage was more pronounced than on previous clothing, and there was some significant material pull‐in by the bolts on some shots. The rear surface damage varied from 4 to 13 mm, with a mean of 8.5 mm in diameter. The 8 mm front surface hole created a 13 mm rear surface hole and experienced a lot of material pull‐in into the wound itself, shown in Figure 11. The final series, hoodie/polo shirt, only had three bolts fully penetrate both clothing layers and the gelatine block, but all the bolts made an impression on the clothing and even the gelatine block in some cases but just could not fully penetrate. There were only three exit holes in the polo, which were all approximately 5 mm in diameter. However, there were more entrance wounds on the polo. These extra front surface impact points of damage created small peaks in the back of the polo where the bolt had pushed the material into the gelatine block but ultimately not penetrated it, leaving the small peaks to solidify from the contact with the gelatine. The shot that penetrated in the first half of this series, 5–2, had red fibers from the hoodie around the entrance of the hole in the polo, but no fibers were carried through into the wound from this shot. There were also two indents on the front of the hoodie from shots that pushed into both layers of clothing but could not penetrate the gelatine block. The second half of the series showed five front surface impact damage holes in the back of the hoodie, but only two go on to penetrate the polo and gelatine block. High‐speed video footage was able to record the effect of the bolts that impacted both hoodie and polo but did not penetrate the block.

## 4 DISCUSSION

### 4.1 Penetration data

As expected, the ballistic gelatine was penetrated by all 10 bolts fired at it. A mean penetration depth of 15.85 cm showed that the bolts were almost all fully inside the gelatine block. This demonstrates how flesh, as the gelatine is there to represent, provides little protection against a crossbow bolt. High‐speed footage shows the ease with which the bolt penetrates the bare gelatine block, although the bolt is shown entering the block before eventually being pulled back a couple centimeters and arresting. This seems to be a result of the convex impact pattern that can be seen when the bolt enters the block. During elastic recovery of the gelatine block, the bolt is then pulled rearwards. When this result is compared with the T‐shirt series (Series 2), it is clear something simple like a T‐shirt can make a difference in penetration reduction. This series had a mean penetration depth of 12.9 cm, 2.95 cm less than that for the gelatine block. Although relatively small, this could be the difference between a vital organ being penetrated or avoided. Statistical t‐test (Table 3) at the 0.05 significance level showed a significant difference between mean penetration values for the gelatine block and T‐shirt series. Further comparison can be made with the polo shirt series, with a mean penetration depth of 14.1 cm. This value was again less than the gelatine block, which was also shown to be significantly different, but slightly more than the T‐shirt series 12.9 cm. However, there was no significant difference between the mean penetration depths of the T‐shirt and polo shirt, meaning these garments do not statistically provide any more protection than the other. Despite this, the two garments respond differently to the crossbow bolts.

**TABLE 3:** Statistical T‐test data (0.05 significance level).

Combination	Observed value	Critical value	Significant difference
Gelatine block vs. Polo shirt & hoodie	10.8860	2.23	Yes
Gelatine block vs. T‐shirt	7.3659	2.11	Yes
Gelatine block vs. T‐shirt & hoodie	7.2000	2.16	Yes
Gelatine block vs. Polo shirt	3.9158	2.11	Yes
T‐shirt vs. Polo shirt	1.9885	2.12	No

Aside from mean penetration data, the fact that not all bolts penetrated both hoodie series shows that thicker layers provide more protection. A mean penetration depth of 12.42 cm for the second half of series 3 showed a further reduction in penetration, albeit only marginal. Mean penetration values for this series (series 3) and the gelatine block were significantly different again, though no significant difference existed between the values for this series and just the T‐shirt (series 2). This is potentially because the mean for series 3 is calculated from only five values, compared with the 10 values for the T‐shirt series (Series 2). Mean penetration for the second half of series 5 was 12.3 cm, which again showed a further reduction in penetration from the previous series. It is important to note this value was calculated with two values, as only two out of five bolts in the series penetrated. Mean penetration values for series 5 and the gelatine block were significantly different again, with the observed value being the highest in the study. These results indicate that overall clothing does have a reducing effect on the penetration capability of crossbow bolts, with the statistical data showing an increase in observed value as the layers or thickness of clothing increase.

A penetrating (projectile impacts target with no residual velocity) projectile transmits more energy to surrounding tissue than a perforating one (projectile travels through a target with a residual velocity), and the extent of the tissue damage depends on the impact velocity of the missile [15]. While crossbow bolts have the capacity to be lethal, in certain circumstances, for example, wound location and availability of medical intervention, these can be survived. The study by Besler et al. reports on a man who attempted suicide using a pistol crossbow [31]. A field tip bolt similar to those in this study was used and penetrated the left thorax and projected to the base of the heart. The bolt was eventually removed, and the man survived. Other reported cases of crossbow injury survival have involved the use of field tip bolts [32, 33, 34, 35], although injury by a broadhead does not mean certain death [22]. Besler notes that this is likely due to the morphology, as a field tip lacks the razor effect of the broadhead, thus causing no incising injuries and even in cases where the patient suffers injury to the heart, brain, or spinal cord, they have survived [31, 33, 34, 35]. Krukemeyer et al. note in their study that due to the elasticity of the soft tissue, it is common for this tissue to effectively bounce back into place and surround the bolt, causing it to act as an incomplete tourniquet and potentially prevent major hemorrhage [36].

### 4.2 Bolt inspection

With regards to the crossbow bolts, there was only one out of 50 that sustained damage, and this was considered minor. As a result, there were no pieces of broken bolt inside any of the wound tracts. In terms of relevance to the forensic investigator, it is seemingly quite unlikely to find any broken pieces of bolt inside the wound tract and therefore hard to link an injury to a crossbow attack through this method. However, over half of the bolts (60%) featured fibers from their associated clothing targets. If, for instance, the bolt is removed from the injury while containing fibers from the victim's clothing, it could be potentially easier for an investigator to link the bolt to the victim through fiber analysis. It is important to note, however, that some styles of clothing are more prone to shedding or distributing fibers than others. This is demonstrated, whereby the polo shirt and hoodie were more prone to shedding fibers compared with the T‐shirt. As highlighted in the results, all bolts fired during the hoodie/T‐shirt series featured a distinct red fiber on the tip. It is unknown as to why this trend arose, but it is likely that it is to do with what has just been discussed in terms of fiber shedding and the fact that the hoodie is fleeced, providing the bolt with a more open layer to pull fibers from. Although not present in this study, the apparent retention capability of the bolt gives potential for blood to be left behind as well. This could be further used to link the victim to the bolt. Furthermore, it is possible that the bolt could retain fingerprints as well from being loaded, with Downs et al. suggesting effort should be taken to preserve possible latent prints [37].

### 4.3 Wound tract inspection

The wound tract inspection revealed some interesting insight, although not as high as the previous bolt fiber percentage. Approximately 28% of the wound tracts contained some type of foreign object, with the majority being clothing fibers, but some appeared to contain an unknown, orange‐tinged stain. It is currently unclear as to the origin and thus requires further study. It is unlikely that most victims will be wearing clothing in a similar new and clean state to that used in the study, and it may instead be dirty or sweaty, for example, which increases the chance of bringing other foreign objects into the wound tract and ultimately the body. It is important to note again, as with the previous bolt inspection, that the type of clothing is a key factor in terms of fiber shedding. There were no T‐shirt fibers reported in either of the two series' wound tracts, and only a couple of tracts contained the red hoodie fibers. It was again the black polo shirt fibers that featured the most in the wound tracts, as they did on the bolts.

### 4.4 Clothing damage

**FIGURE 12:**
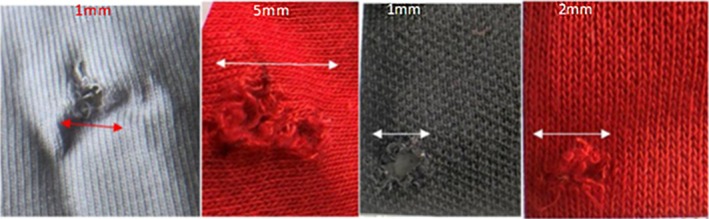
Puncture damage shown on clothing from each series, from L‐R: T‐shirt (series 2), T‐shirt and hoodie (series 3), polo shirt (series 4), and polo shirt and hoodie (series 5) on exit side.

The analysis of the damage sustained by the clothing gives some indication of their failure patterns and how they reacted to the crossbow bolts. It also helps to give some insight to the previous sections detailing fiber distribution. It is important to note, firstly, though, the morphology of the damage. The clothes sustained puncture damage from the crossbow bolts, as explained at the start of this paper. This is a result of the bolt pushing into the garments and penetrating through, tearing and shearing the yarn. This, in turn, created the holes in the garments, with irregular yarn ends and textile distortion that can be seen in all the clothing that was impacted, shown in Figure 12. As described by Robertson et al, the holes seemingly disappear when handled and can only be indicated by frayed yarn ends [25]. While this is not attributed to crossbow holes, the principles and observations from this study can be applied herein. This was especially the case with the front surface impact point on the hoodie. In terms of relevance to a forensic investigator, the appearance of the damage holes in the garments means that it would be sensible to rule out a stab wound, which might be different if the study had used broadheads. While this would be difficult to confirm without further study, MacPhee showed that clothing damaged and its associated patterns were directly affected by the shape of the arrowhead used [24], making it possible to differentiate the arrowhead types from each other when they have a distinct construction.

It is apparent why there can be some misinterpretation between bolt wounds and wounds formed from bullets, stab, and cut, due to the similarity in damage profile. Although from the results of this study, it seems that holes left in clothing because of the bolts could be smaller than those left by bullets, given the bullets larger diameter and faster velocity, while those caused by stab or cutting can vary based on the weapon utilized. Further work, however, is required to confirm this.

The most distinct and extensive damage was that featured on the polo shirt. There were distinctive puncture damage holes, with material pushed in from bolt entry as well as the signature yarn pull‐out. It is likely the holes were created as a result of tension upon impact, compared with a cutting effect as these field tip bolts do not feature the same sharp edges, like broadhead bolts. The entrance holes created in the polo shirt were the widest out of all the series, all being 5 mm except one that was 8 mm. This gives an indication as to the strength of the fabric. It is made from 100% cotton and likely a woven structure. Due to its intended purpose, the textile is a waffle‐like structure that allows for breathability. This looser weave is perhaps why the bolts created the biggest holes.

When comparing the previous data with the T‐shirt, there are some differences between the two. The T‐shirt is constructed with 95% cotton and 5% elastane; this provides the T‐shirt with more elasticity than the polo shirt, allowing for a slightly longer time before failure and potentially explaining why the mean penetration depth was slightly less than for the polo shirt. MacPhee et al. highlight the characteristic of higher elastane levels in clothing providing longer failure times [24]. The T‐shirt again features the same puncture damage holes, but these were all smaller than the polo shirt, ranging from 3 to 4 mm. It is likely that the T‐shirt has a tighter knit than the polo shirt, which is why there was a smaller mean penetration depth, although it is important to note that there was no significant difference between the two mean values.

**FIGURE 13:**
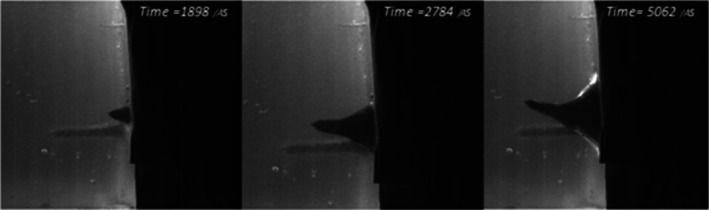
Stills of high‐speed video of series 3, showing from L‐R: First impact of crossbow bolt, the bolt then pushing the clothing layers to its maximum tension level, the bolt finally penetrates the clothing layers and pushes into the gelatine block.

Given how the T‐shirt reacted to the crossbow bolts, it would be logical to assume that adding a hoodie in front would further reduce the penetration capacity. This was only partly the case, producing a smaller mean penetration depth, but it proved not to be significantly different from just the T‐shirt. The hoodie was made from 50% cotton and 50% polyester, which means it is not the highest quality, but polyester does have a reasonably tight knit. However, polyester fleece is known to shed fibers too, which was evident on the bolts and in some of the wound tracts. High‐speed footage of the third shot from the first half of the series shows the hoodie and T‐shirt being forced into the gelatine block by the bolt before being eventually penetrated. Shown in Figure 13, the resistance of the clothing can be seen before reaching its failure point. The bolt is shown being forced up, which seems to reduce the penetration capability compared with a bolt that impacts the clothing straight on. Although the bolt penetrated, the resistance of the clothing will potentially reduce the damage done, resulting in a shallower wound tract (7.5 cm) and probable bruising.

**FIGURE 14:**
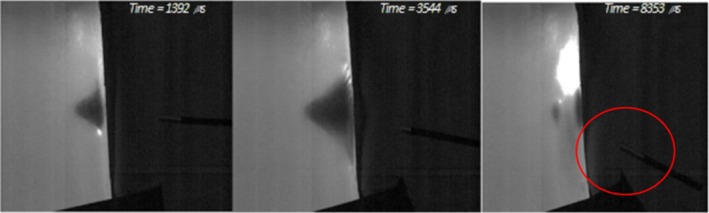
Stills of high‐speed video of series 5, showing from L‐R: First impact of crossbow bolt, the bolt then pushing the clothing layers to the apex of impact, the bolt cannot ultimately penetrate the clothing and is pushed back out and falls to the floor.

As demonstrated by the statistical data, the polo shirt by itself performed the worst out of the clothing combinations compared with the gelatine block. When paired with the hoodie, it was most effective at protecting against penetration. The three shots that did penetrate again featured the same puncture damage characteristics as the other clothing. However, shots that did not fully penetrate also features some puncture damage, where the bolt has penetrated through the hoodie but ultimately not through the polo shirt. This can be seen in Figure 14. The bolt impacts both the clothing layers and pushes into the gelatine block but lacks the capability to overmatch the resistance of the clothing layers. The impact can then be seen receding as the bolt is eventually pushed back out of the clothing and onto the floor. This non‐penetration event is featured in a similar fashion in the work by Wightman et al., where air rifle pellets did not have enough energy to penetrate the clothing and gelatine block, resulting in imprints left in the block [38]. Wightman also notes the effect the pellets have on the clothing, detailing how the pellets formed cup‐like shapes where the material was strained but not broken [38]. This is similar to the small peak‐like indents left in the polo shirt, where the bolt did not manage to penetrate the clothing. It is important to note that this footage is from the second half of the series when the hoodie was double layered, although the results show that only two bolts penetrated when the hoodie was single layered. This shows that this combination for the majority of the shots provides too much resistance for the bolts to push through, from this crossbow at least. It remains to be seen what effect a crossbow with a larger draw weight would have.

The protection mechanism of the clothing can be broken down. From literature, clothing has been shown to cause flight properties of bullets to alter before impacting the body, thus increasing potential risk [26, 27, 39]. This long impact time allows for the transfer of kinetic energy to the fabric, dissipating it and thus rendering the bullet stationary [40]. This principle can be roughly applied to this clothing combination, where the bolt was witnessed to be in contact with the fabric before arresting. The contact time between the bolt and T‐shirt, for example, is not long enough for the kinetic energy of the bolt to be transferred and dissipated, resulting in penetration of the T‐shirt. This concept ties in with what was previously discussed, concerning the strength of weave/knit and yarn thickness [25]. Clothing with a stronger or tighter weave/knit can absorb more energy due to the support of the surrounding fibers, thus requiring a higher amount of energy in order to fail [24]. There is also the potential that the hoodie/polo shirt combo was able to prevent/reduce penetration based on the theory in the MacPhee et al. study, as it was draped loosely [24]. This is also supported by the study by Wightman et al., who state that as looser clothing is able to move more, it can absorb more energy compared with taut clothing, which can absorb less energy before being penetrated [38].

Despite this shot not penetrating, it is important to note the impact that the shot would still have on an individual. The bolt has pushed into the flesh by approximately 4 cm. Although this value may not seem large, it still has the potential to cause some internal damage that may not be visible, like an arrow wound. While not entirely the same circumstances, the high‐speed footage from the shot is reminiscent of a process known as behind armor blunt trauma (BABT) [41]. The deformation of the armor effectively punches the wearer, leading to injuries that are associated with blunt force trauma such as internal bleeding, contusions, and hematoma [41]. As noted in the introduction, a crossbow bolt travels at substantially slower than a handgun bullet; thus, the extent of the injuries sustained as a result of this “non‐penetration” impact are not likely to be as severe as seen in a gunshot wound. It would be beneficial, however, for a medical professional to know the potential implications of somebody who has been shot with a crossbow that did not penetrate their skin.

## 5 LIMITATIONS

Potential limitations encountered in this study are concerned with the placement of the clothing on the gelatine block and the firing of the crossbow bolts. As highlighted in the research by MacPhee et al., the clothes that were tighter around the gelatine block were penetrated more easily, generally because there was less surface area to catch and dissipate the energy of the bolt [24]. This could apply to some of the series in this study. The nature of the experiment meant leaving the bolts in after firing; the clothing could have experienced a pinning effect whereby the more bolts that had been fired into it, the tighter the material became. This could have made the T‐shirt tighter and left it open to being more easily penetrated. There are no trends of significance in the dataset to fully support this, but it is something to be aware of. Another limitation of this study is the exclusion of a skin layer or skin analogue. As the gelatine block is meant to represent flesh, it is unknown how or if a skin analogue would affect the results. Skin has a different morphology and function than flesh, acting as a natural barrier. There is potential for the results to change if a skin layer were included in this study and should be explored as part of future works.

## 6 CONCLUSION

The focus of this study was to see the impact effect of crossbow bolts on different types and combinations of clothing, using ballistic gelatine as a flesh analogue. All bolts were able to penetrate single‐layer clothing and the non‐clothed gelatine block, with nine penetrating the hoodie/T‐shirt combination and only three for the hoodie/polo shirt combo. It was proven that clothing provides a significantly higher level of protection compared with the non‐clothed gelatine block. The thicker the clothing layer combination, the more protection against crossbow bolts than the single layers of clothing. Aside from the gelatine block, the polo shirt had the highest mean penetration depth out of all the clothing used. Inspection of wound tracts and individual bolts revealed the presence of clothing fibers, especially in the series involving the polo shirt and hoodie. Clothes made with a looser weave or from materials such as polyester were more prone to shedding fibers. The presence of these fibers may aid investigators in being able to link clothing or weapons with victims and/or perpetrators. High‐speed footage revealed potential internal injuries that were not observed during the experimental procedure due to the crossbow bolt impacting the target and being arrested before rebounding out of the target or before receding with no penetration. In turn, contusions or internal bleeding may occur as a result. While the results of this study provide insight into how clothing reacts against crossbow bolts, it would be beneficial to further this study by comparing similar bolt configurations delivered between crossbow and bow and arrow to determine if there are any notable differences.

## CONFLICT OF INTEREST STATEMENT

The authors declare no conflicts of interest.

## Data Availability

The data that support the findings of this study are available from the corresponding author upon reasonable request.
